# Sensitivity Optimization and Experimental Study of the Long-Range Metal Detector Based on Chaotic Duffing Oscillator

**DOI:** 10.3390/s22145212

**Published:** 2022-07-12

**Authors:** Timur Karimov, Olga Druzhina, Valerii Vatnik, Ekaterina Ivanova, Maksim Kulagin, Veronika Ponomareva, Anzhelika Voroshilova, Vyacheslav Rybin

**Affiliations:** 1 Youth Research Institute, St. Petersburg Electrotechnical University “LETI”, 5 Professora Popova St., 197376 Saint Petersburg, Russia; osdruzhina@etu.ru (O.D.); mvkulagin@etu.ru (M.K.); 2 Department of Computer-Aided Design, St. Petersburg Electrotechnical University “LETI”, 5 Professora Popova St., 197376 Saint Petersburg, Russia; vmvatnik@stud.etu.ru (V.V.); emivanova@stud.etu.ru (E.I.); veponomareva@stud.etu.ru (V.P.); 3 School of Public Administration and Entrepreneurship, Institute of Economics and Management, Ural Federal University Named after the First President of Russia B.N.Yeltsin, 51 Lenina Ave., 620075 Yekaterinburg, Russia; a.i.voroshilova@urfu.ru

**Keywords:** chaos theory, chaotic sensor, Duffing oscillator, inductive sensor, proximity sensor

## Abstract

Sensors based on chaotic oscillators have a simple design, combined with high sensitivity and energy efficiency. Among many developed schemes of such sensors, the promising one is based on the Duffing oscillator, which possesses a remarkable property of demonstrating chaotic oscillations only in the presence of a weak sine wave at the input. The main goal of this research was to evaluate the maximal sensitivity of a practically implemented metal detector based on the Duffing oscillator and compare its sensitivity with conventional sensors. To achieve high efficiency of the Duffing-based design, we proposed an algorithm which performs a bifurcation analysis of any chaotic system, classifies the oscillation modes and determines the system sensitivity to a change in different parameters. We apply the developed algorithm to improve the sensitivity of the electronic circuit implementing the Duffing oscillator, serving as a key part of a three-coil metal detector. We show that the developed design allows detecting the presence of metal objects near the coils more reliably than the conventional signal analysis techniques, and the developed detector is capable of sensing a large metal plate at distances up to 2.8 of the coil diameter, which can be considered a state-of-the-art result.

## 1. Introduction

One of the relatively new directions of sensor design, driven by progress in the field of nonlinear dynamics, is the application of chaotic oscillators for sensing and measurements. An attractive property of chaotic systems for sensing applications is that such systems are highly responsive to small changes in parameters. Application of nonlinear oscillators allows increasing the sensitivity and range of sensors [1], linearizing their response [2,3] and providing several other useful properties; in particular, such sensors were shown to be good candidates for the weak and noisy signals detection [4,5].

The Chua circuit and the Duffing oscillator are often used for constructing chaotic sensors. A remarkable feature of these systems is a double-scroll attractor. In recent work by Wu et al. [6], the Duffing oscillator was used to build a sensor for pipe structure defect detection. Authors show that their approach may significantly enhance the sensitivity of minor defect detection in pipe structures. Zhao et al. [7] applied a Duffing oscillator to measure weak infrared radiation intensity. This approach allowed keeping measurement error below 16.1% at a signal-to-noise ratio of −67 dB. Korneta et al. [2] proposed a noise-activated DC voltage signal sensor based on the Chua circuit. The detection strategy is based on the monitoring of switching between attractor scrolls. The switching process is activated by the noise added to a measured signal. The designed sensor performed notably well at noisy DC signals, as the noise is inherent in its functioning.

The iconic series of papers by W. Hu and co-authors [8,9,10] describes the Duffing oscillator-based metal detector. Using the numerical simulation, the authors show that the sensitivity of the proposed design is extremely high compared to classic sensors: the signal amplitude threshold which can be detected was evaluated as $10^{-9}$ V (−180 dB) [8]. Hu claims [9] that in an experiment with 30 mm diameter sensitive coils the metal detector managed to discover iron particles of 1.5 mm and aluminum particles of 2 mm in diameter, respectively.

One of the problems in designing sensors based on chaotic oscillators is the extraction of a measured value from a chaotic signal. Such methods as the attractor geometric dimensions analysis [3], the use of recurrence quantification analysis (RQA) [11], the number of orbits calculation [12], feature extraction from attractor with the subsequent interpretation by look-up table (LUT) or machine learning [13] and other methods were proposed. The convenience of the Duffing and Chua oscillators lies in the fact that the moments when their attractor scrolls are switching can be interpreted as spikes of neurons [14], which make it possible to apply various effective methods for neuron activity analysis, for example, inter-spike intervals distribution visualization. The spike analysis method proved to be very effective for sensing in our previous study [15]: we implemented a single-coil metal detector based on a Sprott Case N chaotic oscillator with spiking behavior and demonstrated that the developed metal detector has a continuous response depending on the distance to the target and the target material (steel, aluminum or brass/copper). We compared the developed chaotic sensor with the sensor based on a harmonic oscillator and found that the operating range of the chaotic sensor is at least 20% larger than that of the harmonic one.

Meanwhile, each of the detection methods requires the choice of the appropriate operating mode for the chaotic system. For example, the attractor geometric dimensions analyzing [3] requires stable chaotic oscillations, while the analysis of switching between scrolls [14] is more efficient at the boundary of the periodic and chaotic regimes. Searching for bifurcation parameters providing the highest sensitivity of a chaotic sensor is a non-trivial and little-explored problem. Its complication is that many chaotic oscillators have several zones of continuous chaos and mode switching, so the sensor designer has to make a choice about which zone should be used.

Based on these assumptions, we concluded that the results achieved by W. Hu in his experiments [9] did not reveal the full potential of the proposed metal detector and built our own version of this development. The contribution of the current study is as follows:

1. We propose a quantitative estimation of the chaotic oscillator’s sensitivity and based on it, present an algorithm for searching for their most sensitive regimes. This information is of vital importance when constructing chaotic sensors.
2. Using the developed approach, we study the sensitivity of the electronic circuit implementing the Duffing oscillator. Numerically, we find a parameter set at which the circuit most rapidly switches from periodic to chaotic mode and verify our findings experimentally.
3. We apply the Duffing circuit in the metal detector proposed by W. Hu et al. This detector consists of three coils, a differential amplifier, the Duffing oscillator and a detector. We propose our own designs of differential amplifier and detector circuits, as well as the numerical detection algorithm by analyzing the Duffing oscillator state variables by counting the switching rate between attractor scrolls. This method of oscillations’ quantification has never previously been applied to constructing the sensor based on the Duffing oscillator.
4. In a well-controlled experiment, we study the sensitivity and accuracy of the developed metal detector by finding limit distances at which the detector is capable of sensing the metal plates of different materials and sizes. Such tests had not yet been performed for the metal detector of the proposed design. Then, we compare the obtained results with available data on inductive sensors and metal detectors.

The paper is organized as follows. In Section 2, we described the algorithms and the circuit design. In Section 3, numerical and experimental results are given, including phase portraits, bifurcation diagrams and sensitivity analysis. Section 4 briefly discusses obtained results and their practical value. Section 5 concludes the paper. Additional photographs of the experiments and plots are given in Appendix A.

## 2. Materials and Methods

### 2.1. Sensitivity Optimization Algorithm

The sensitivity σ of the system is the derivative of the metric *M* with respect to the parameter. In the case of a chaotic system, it can be defined as a derivative of a mean value, or a root-mean-square (RMS) value, for a bifurcation parameter value *p*:

(1) $$\sigma =\frac{\Delta M}{\Delta p}.$$

Since a variety of operating regimes is inherent to chaotic systems, their sensitivity can vary greatly depending on the parameter sets.

The determination and classification of oscillation regimes is an integral part of chaotic systems study. For these purposes, an algorithm was developed. The classification of the chaotic oscillation regimes consists of the following steps:

1. Calculation of the largest Lyapunov exponent (LLE) using a method presented in [16]. In the study of chaotic systems, it is the most frequently used indicator of chaos presence, which allows for accurately distinguishing the chaotic mode from the periodic.
2. Plotting a bifurcation diagram (BD) with respect to the varying parameter. Bifurcation diagram analysis allows for visually presenting the system dynamics, including sharp mode transitions, as well as counting the number of periodic orbits.
3. Searching for bifurcation diagram areas with higher sensitivity. This requires tracking the rapid changes in the chaotic system dynamics. In this stage, we apply a low-frequency bandpass filter to decrease the non-chaotic signal amplitude compared to the chaotic signal. Then we calculate the difference between adjacent average signal values and find the derivative with respect to the varying system parameter. After applying the algorithm, we obtain a plot of the system sensitivity with bursts at the points of oscillations mode changing.
4. For segments with a zero Lyapunov exponent, i.e., periodic oscillations, the number of periodic orbits is counted. The field of the bifurcation diagram is divided into a matrix of bins of size *M* × *N*, where *M* is the specified number of bins and *N* is the number of parameter values. For each value of the bifurcation parameter, we check whether a point of the bifurcation diagram belongs to the bin corresponding to a certain range of state variable amplitude values. Each appearance of a bifurcation diagram point in the bin is counted. Then, the periodic orbits are calculated according to the following algorithm:
  - If the first (upper) bin contains points and the second (lower) bin is empty, then consider it as a separate orbit;
  - If the bin in the middle is full, and the next (lower) bin is an empty one, then consider it as a separate orbit (in the case of several filled bins located next to each other, consider them all belonging to the same orbit);
  - If the last bin is full, and the bin before it (upper bin) is empty, then consider it a separate orbit.

**Figure 1:**
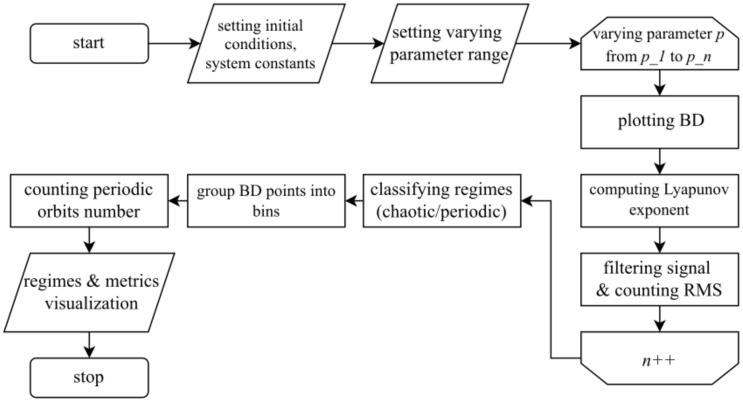
The block diagram of an algorithm for oscillator sensitive modes searching.

The complete sensitivity search algorithm is presented in Figure 1.

The results of the algorithm application are given in Section 3.1.

### 2.2. Metal Detector Based on the Duffing Oscillator

#### 2.2.1. The Duffing Oscillator

**Figure 2:**
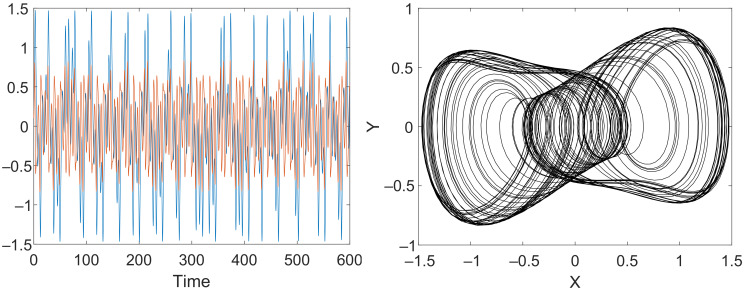
Time series of the classical Duffing-Holmes system and corresponding phase portrait.

The Duffing, or Duffing–Holmes oscillator, is a well-known chaotic system exhibiting chaotic behavior when a sine wave of particular amplitude and frequency is fed to it [17]. It is described by the second order equation:

(2) $$\ddot{x}+b\dot{x}-x+x^{3}=a\sin\omega t,$$

which can be presented as a system of equations:

(3) $$\begin{array}{c} \dot{x}=y\\ \dot{y}=F\left(x\right)-by+a\sin\omega t, \end{array}$$

where $F\left(x\right)=x-x^{3}$. In (2) and (3), *b* is the damping coefficient, while *a* and ω are the amplitude and radial frequency of the input sine wave, respectively. Waveform and phase portrait of the solution of the Duffing system are presented in Figure 2.

#### 2.2.2. Conceptual Design

**Figure 3:**
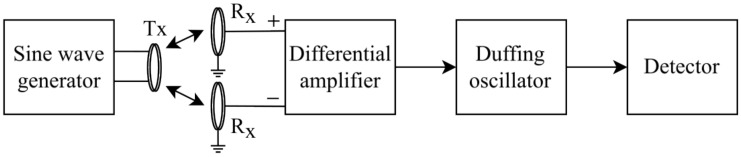
Schematic of the Duffing oscillator-based metal detector.

The basic concept was proposed by Hu and Liu in 2010 [8]. The metal detector consists of the following blocks: a generator of harmonic oscillations driving the transmitting coil; two receiving coils connected to a differential amplifier; the Duffing oscillator, receipting the differential voltage between sensitive coils and the detector forming information about the presence or absence of a metal object near the coils by the analysis of oscillations in the Duffing oscillator, see Figure 3.

The system works as follows. The sine wave generator creates harmonic electromagnetic oscillations in the transmitter (*Tx*) coil. Both receiver (*Rx*) coils pick up this signal; due to the imperfectness of geometrical and electrical parameters of these coils, the difference in signals of *Rx* coils $\Delta V=V_{Rx1}-V_{Rx2}$ always exists. The differential amplifier (DA) reads the difference between these signals ΔV and amplifies it many times (e.g., 50), as ΔV has a small amplitude (of the order of millivolts) and is noisy due to electromagnetic interference. In addition, ΔV is filtered so that only frequencies in the range corresponding to the frequency of the sine wave generator remain. The signal prepared in this way is fed into the Duffing oscillator. The overall system must be configured in such a way that in the idle state, the Duffing oscillator demonstrates periodic oscillations, and when a metal object appears near the coils, it switches to a chaotic mode. To configure the system, the threshold voltage $V_{0}$ of switching the Duffing oscillator from periodic to chaotic mode is determined, and the gain of the differential amplifier is adjusted so that in the absence of metal objects, the output voltage amplitude of the differential amplifier does not exceed $V_{0}$. To define in which oscillation mode, periodic or chaotic, the Duffing oscillator currently operates, the detector is used, which can be based on various principles of operation, for details see Section 2.2.4.

#### 2.2.3. Circuit Design

In this section, two parts of the metal detector are considered: the differential amplifier and the Duffing oscillator.

**Figure 4:**
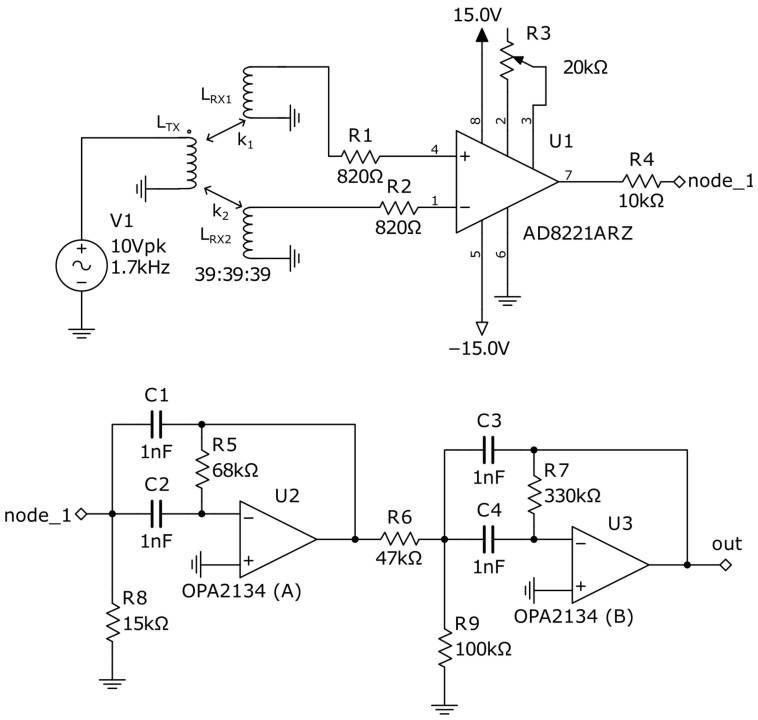
Differential amplifier and an output filter bandpassing frequencies between 1 kHz and 10 kHz with 20 dB decay in rejected bands, which had been implemented in our study on a circuit board. The generator V1 desirably has a tunable frequency.

**Figure A1:**
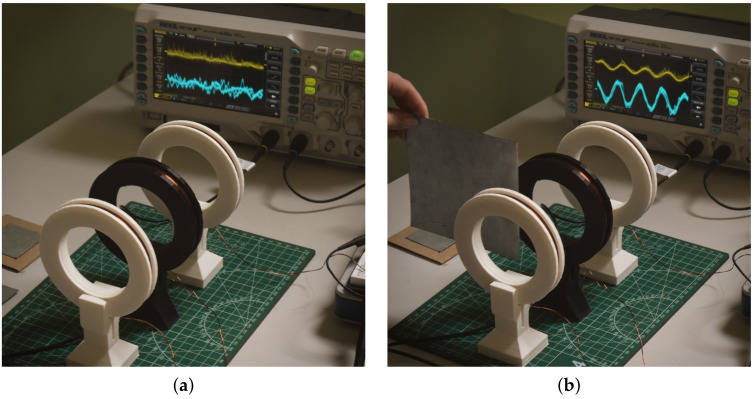
Output of the differential amplifier: (**a**) in the absence of any metal object, and (**b**) in presence of a steel plate put between transmitting (black) and one of the receiving (white) coils. The yellow line is the instrumental amplifier output, and the blue line is the output of the filter. The last signal is used to drive the Duffing oscillator.

Figure 4 presents a differential amplifier based on the instrumental amplifier AD8221 (Analog Devices, Norwood, MA, USA), followed by a bandpass filter allowing us to cancel a DC component of the signal, as well as a high-frequency noise. Such a filter allows improving the sensitivity of the metal detector. The variable resistor $R_{3}$ allows adjusting an amplitude of the signal driving the Duffing oscillator. In Figure A1, the operating principle of a DA is explained.

The authors of the current metal detector concept, Hu and Liu, also developed an electronic circuit implementing the Duffing oscillator [4]. In a computer simulation, the proposed circuit was found to be suitable for detecting weak high-frequency signals, as well as filtering high-frequency noise. However, the circuit design is rather complicated and costly, e.g., it involves four analog multipliers.

**Figure 5:**
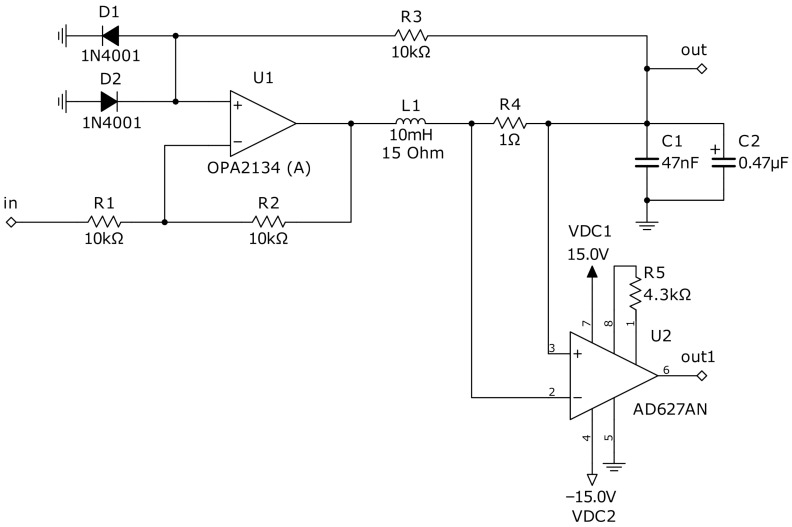
Circuit implementation of a Duffing oscillator with diodes and inductive coil used in our study. The instrumental amplifier U2 allows observing the current in the coil L1, which is one of the state variables. Output *out* allows observing the voltage state variable, and *out1*—the current. In the practical design, *out1* is not required, as it is used only to obtain a two-dimensional phase plot.

Thus, we found in [17] a simple and elegant electronic Duffing oscillator containing only one operational amplifier and a couple of antiparallel diodes forming the non-linearity. The proposed circuit is presented in Figure 5. With the indicated components values, the chaotic mode is established with the following parameters of the input sine wave: f=1700 Hz and A=240 mV.

This circuit implements (2) and (3) electrically and is described by the following equations [17]:

(4) $$\begin{array}{cc} C\frac{dV_{C}}{dt} & =I_{L}\\ L\frac{dI_{L}}{dt} & =F_{E}\left(V_{C}\right)-I_{L}R+A\sin\left(2\pi ft\right) \end{array}$$

where $V_{C}$ is the voltage at the capacitor $C=C_{1}+C_{2}$ (and the main output of the circuit) and $I_{L}$ is the current through the inductor $L_{2}$. The nonlinearity $F_{E}\left(V_{C}\right)$ produced by antiparallel diodes is approximately described by a three-segment piecewise linear function:

(5) $$F_{E}\left(V_{C}\right)=\left\{\begin{array}{cc} -\left(V_{C}+kV^{*}\right), & V_{C}<-V^{*}\\ \left(k-1\right)V_{C}, & -V^{*}\leq V_{C}\leq V^{*}\\ -\left(V_{C}-kV^{*}\right), & V_{C}>V^{*} \end{array}\right.$$

For silicon diodes, the value $V^{*}\approx 0.5$ V at I=0.1 mA is relevant. If $R_{1}=R_{2}$, then k=2. Thus, noting $x=V_{C}$ and $y=I_{L}$, (4) and (5) may be rewritten as:

(6) $$\begin{array}{cc} \dot{x} & =C^{-1}y\\ \dot{y} & =L^{-1}\left[F_{E}\left(x\right)-Ry+A\sin\left(2\pi ft\right)\right] \end{array}$$

(7) $$F_{E}\left(x\right)=\left\{\begin{array}{cc} -(x+1), & x<-0.5\\ x, & -0.5\leq x\leq 0.5\\ -(x-1), & x>0.5 \end{array}\right.$$

In (6), $R=R_{L_{1}}+R_{4}$. Expressions (6) and (7) will be used in the further simulations.

#### 2.2.4. Recognition of the Duffing Oscillator Operation Mode

**Figure 6:**
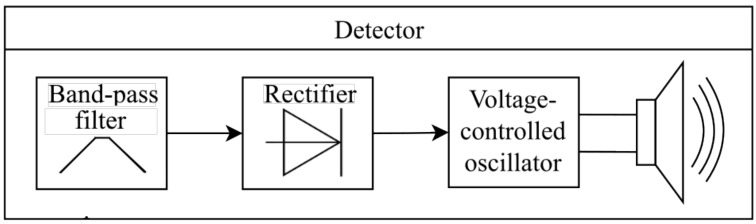
Scheme of the analog Duffing oscillator mode detector.

**Figure 7:**
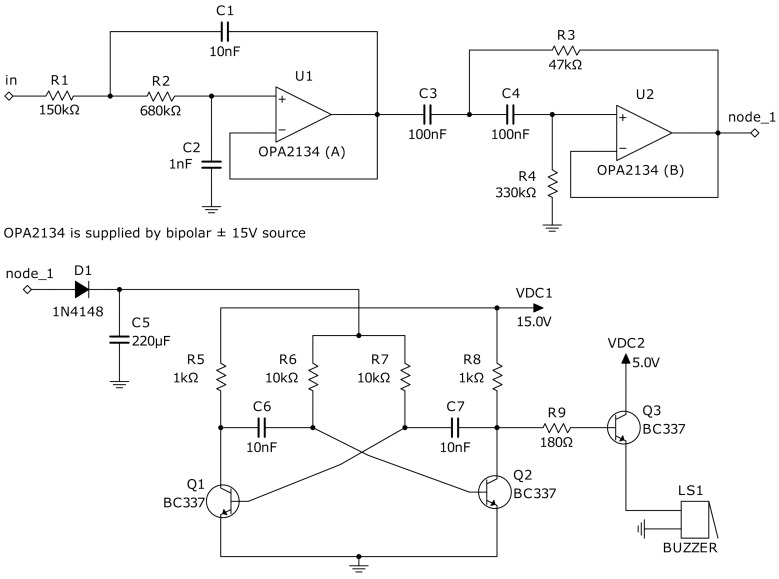
Electrical schematic of the analog Duffing oscillator mode detector. In the absence of chaotic oscillations at the input, it produces a very high tone. In the presence of a chaotic signal at the input, a low scroll-switching rate produces a low tone, and a high scroll-switching rate produces a high tone.

Various types of detectors can be used for the oscillations mode recognition. Hu and Liu [8] proposed to calculate the largest Lyapunov exponent of the output signal, but this algorithm is computationally expensive. As alternatives, the following methods can be proposed: the analysis of the output signal spectrum [18] and the analysis of the switching rate between attractor scrolls [14]. The advantage of the first method is that it can be implemented in a completely analog way. The block diagram of an analog detector is shown in Figure 6. It consists of a bandpass filter, a rectifier, a voltage-controlled oscillator and a loudspeaker or any other suitable indicator, i.e., LED. The filter selects the frequency band from the chaotic oscillations spectrum which is present in the chaotic mode and absent in the periodic one; the idea is described in more detail in [18]. The amplitude of the bandpassed signal is detected by the rectifier, and this rectified voltage serves as a control signal for the audio frequency generator. The operator of the metal detector hears a significant change in tone when the mode of the Duffing oscillator changes. A possible schematic of an analog detector is shown in Figure 7.

The second type of detector converts the signal of a chaotic oscillator into a signal similar to neuron spikes [14], which makes it possible to apply various effective methods to it, for example, analysis of the distribution of inter-spike intervals and classification of the resulting output using machine learning [15]. This method is applicable to digital computers only, but it is simple and provides great capabilities of recognition using machine learning.

**Figure 8:**
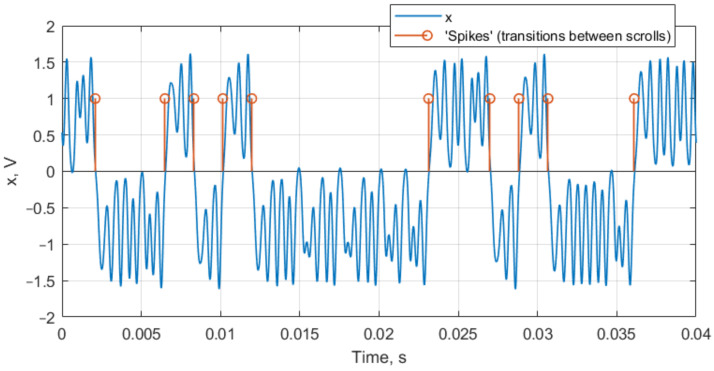
Conversion of two-scroll attractor chaotic system waveform *x* into a spike train.

The Duffing oscillator signal conversion into a spike train is explained in Figure 8.

One of the possible conversion algorithms is presented in [14], though we used another method based on a conventional peak detector. The oscillation mode can be determined from the distribution of spikes:

- When there are no spikes, the oscillator is in a periodic mode.
- When there are very few interspike intervals present, the oscillator is also in a periodic mode.
- The chaotic mode is characterized by a variety of interspike intervals with the presence of rare ones.

An intensity of switching is determined by calculating the number of spikes and is informative as being related to the input sine wave amplitude and frequency.

## 3. Results

**Figure 9:**
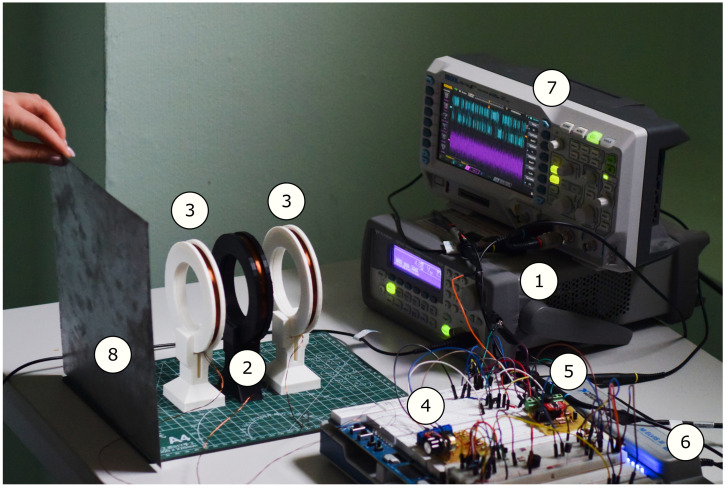
Experimental setup. Here: (1) digital sine wave generator Keysight 33210A, driving the transmitter coil (2); (3) receiving coils; (4) differential amplifier; (5) chaotic circuit implementing the Duffing oscillator; (6) NI ELVIS III power supply and digital data acquisition station; (7) oscilloscope Rigol DS1054Z displaying diff-amp out (violet wave) and the Duffing oscillator out (blue wave); (8) metal plate used as a target. The Duffing oscillator is in a chaotic mode. The coil diameter is 10 cm.

The photo of the Duffing oscillator-based metal detector is presented in Figure 9. The study of the design consisted of the following stages:

  (a) Tuning of the mathematical model parameters to better match the real circuit.
  (b) Using the tuned model, search for the most sensitive regime with the developed algorithm.
Verification of the regime experimentally.
Investigation of the developed detector sensitivity on a set of test targets.
Comparison of the differential amplifier signal analysis by the Duffing oscillator and conventional signal-processing methods.

### 3.1. Numerical Optimization of the Duffing Oscillator-Based Metal Detector System Parameters

To make the process more accurate, we tuned values $V^{*}=0.52$ V and C=630 nF of the model according to the method proposed in [19]. As a result of the sensitivity search algorithm, we found the most rapid oscillation mode transition at A=0.152 V and a frequency of 1700 Hz. Note that only frequencies that are multiples of 100 were considered.

**Figure 10:**
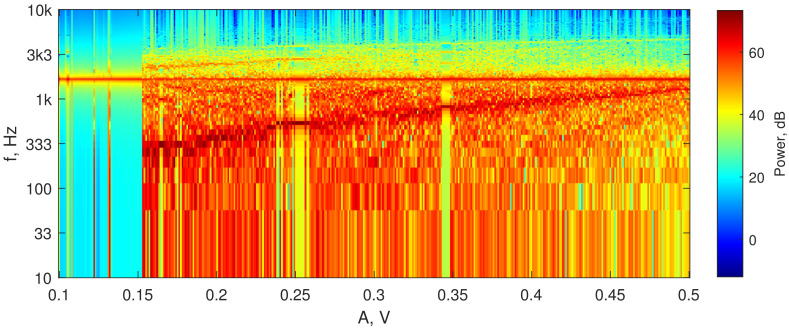
Spectral diagram of the Duffing oscillator fed with input sine wave of frequency f=1700 Hz and amplitude A,V. One may see that that the energy of low-frequency oscillations in the periodic mode is 20 dB lower (turquoise and yellow regions) than in the chaotic mode (red regions). This spectral property is used for constructing an analog oscillations mode detector—see Section 2.2.4.

**Figure 11:**
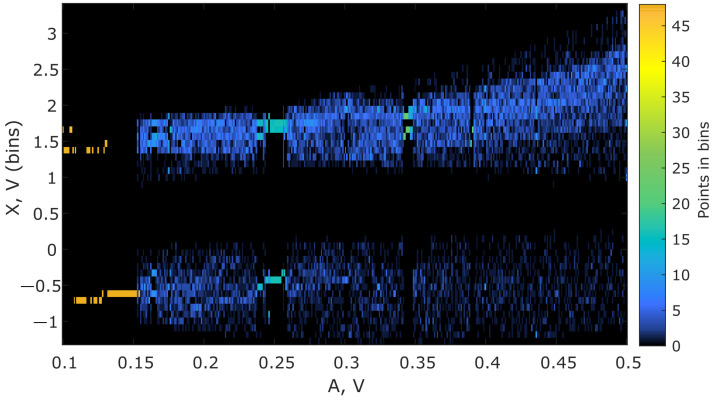
Bifurcation diagram of the Duffing oscillator with points sorted into bins. The field of the diagram is a matrix of bins of size M × N, where M is the specified number of bins, and N is the number of parameter *A* values. Points of a bifurcation diagram in the bins are counted.

**Figure 12:**
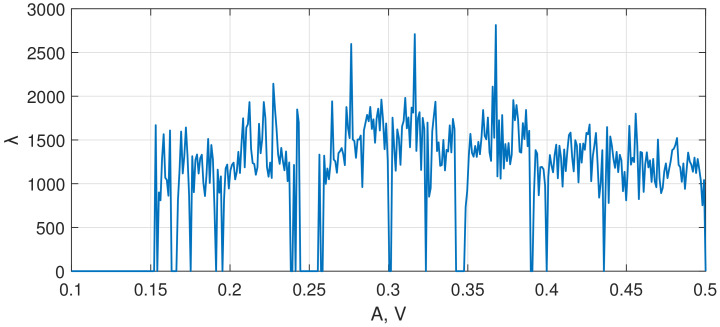
Dependence of the largest Lyapunov exponent (LLE) on the input sine wave amplitude.

**Figure 13:**
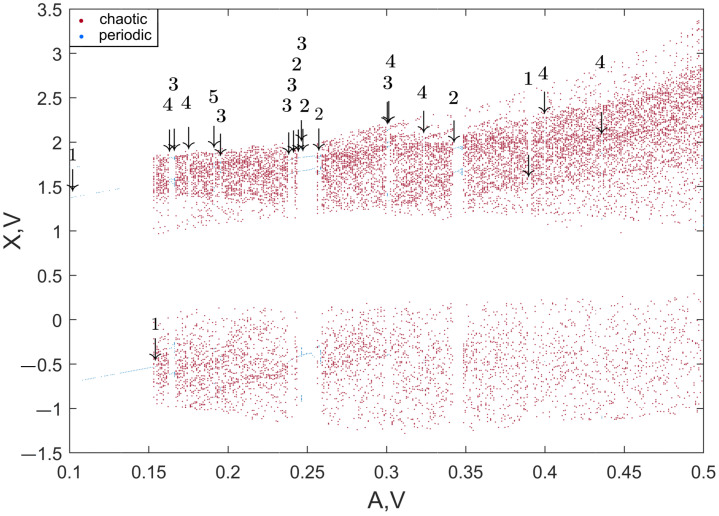
Bifurcation diagram of the Duffing oscillator at 1700 Hz sine wave input with variable amplitude with classified oscillation modes. The numbers indicate quantities of periodic orbits.

To perform the numerical optimization of the developed sensor’s parameters, we applied the algorithm described in Section 2.1. Figure 10 presents the spectral diagram of the Duffing system after applying the lowpass filter, and Figure 11 demonstrates the bifurcation points of the Duffing oscillator sorted into bins. To distinguish between chaotic and periodic regimes, the LLE was calculated (Figure 12). The plot clearly demonstrates the regions of chaotic and periodic behavior. We also plotted the bifurcation diagram, which represents the chaotic oscillations (clouds of points), periodic regimes with one scroll and some bifurcation points where scrolls double (Figure 13). This type of diagram is calculated from sorted BD (Figure 11) and could be useful for determining the number of orbits associated with the value of the measured parameter as proposed by Bin et al. [12]; however, for a given oscillator, this number does not exceed four and practically cannot be used for measurements.

**Figure 14:**
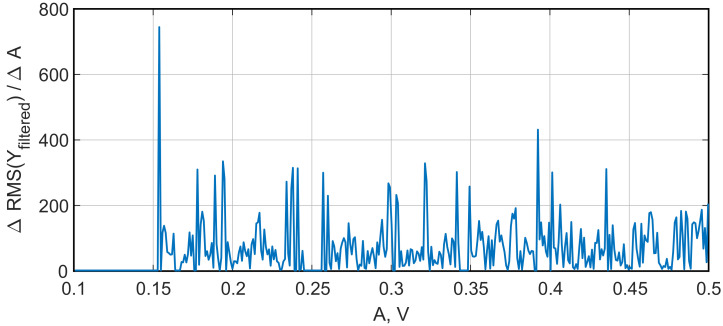
Dependence of the Duffing oscillator sensitivity on the input sine wave amplitude.

As one can see, in the region of A≈0.17 V, the single orbit instantly jumps to the chaotic oscillations; so this is considered to be a point of the sharpest oscillations switching. As a confirmation of this suggestion, the function of systems sensitivity σ was plotted (Figure 14). It is described as follows:

(8) $$\sigma =\frac{\Delta RMS(Y_{filtered})}{\Delta A},$$

(9) $$RMS\left(Y_{filtered}\right)=\sqrt{\frac{1}{n}\left(Y_{1}^{2}+Y_{2}^{2}+\ldots Y_{n}^{2}\right)},$$

where $Y_{filtered}$ is a chaotic signal of the Duffing system after a bandpass filter is applied. Note that in the area of A≈0.17 V, the sensitivity value is the highest, σ = 767. The next most sensitive point is at A≈0.39 V, where the σ is almost half as much, and there is a very narrow region of the periodic regime. We also examined other frequencies with a step of 100 Hz from 1400 Hz to 2300 Hz and did not find any σ>767. Thus, the sharpest switch from periodic to chaotic oscillations of the Duffing system was found both visually and by using the developed software. During further studies, the oscillations with a set of parameters 169 mV and 1700 Hz are considered the most sensitive mode of the Duffing oscillator.

### 3.2. Metal Detector Performance Study

**Figure 15:**
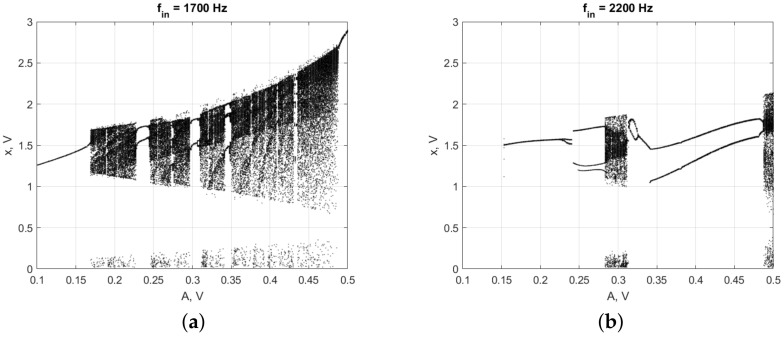
Bifurcation diagrams of the Duffing circuit at different frequencies: (**a**) 1700 Hz; and (**b**) 2200 Hz. We have chosen the frequency 1700 Hz as the sensitivity σ in the sense of definition (1) is higher in this case.

To make sure that the set of parameters found by the algorithm provides the most abrupt switching between periodic and chaotic modes, we plotted bifurcation diagrams of the analog Duffing circuit, see Figure 15. The applied method of circuits bifurcation analysis is presented in [20]. Measurements accuracy is provided by the use of a high-precision measurement station NI ELVIS III with the resolution of 16 bit per ±10 V (ϵ = 0.3 mV) and high precision digital frequency generators with error less than 0.01 Hz.

We found two rapid transitions: at 169 mV and 1700 Hz, close to numerically found, and at 283 mV and 2200 Hz. The first parameter set was used for the further experiments, as the sensitivity in this mode was found to be higher as the model predicted.

**Figure 16:**
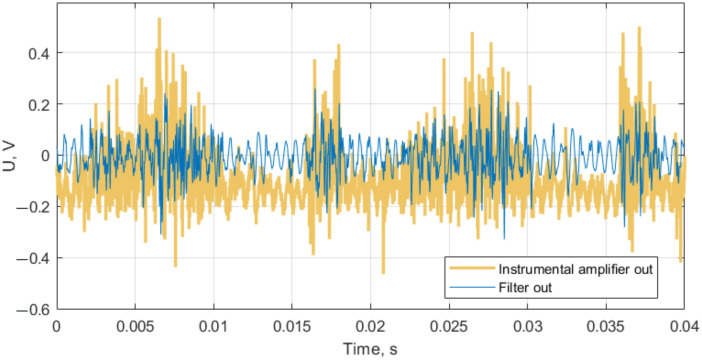
Signal of differential amplifier unit in the absence of the metal object: output signals of two stages are presented.

To confirm the correct operation of the overall metal detector, we have inspected the operation of the differential amplifier and the filter (the schematic was presented earlier in Figure 4). The operation of the DA is illustrated by photographs in Figure A1; it worked as expected, increasing its output amplitude when the metal object moved closer to one of the coils, and decreasing when this object moved closer to another. A recording of the low-amplitude DA signal is presented in Figure 16. The operation of the bandpass filter is also illustrated in this figure. The filter stage cancels DC bias and rejects high-frequency noise as intended.

After that, we performed the experimental evaluation of the metal detector sensitivity.

### 3.3. Investigation of the Developed Detector Sensitivity on a Set of Test Targets

**Figure 17:**
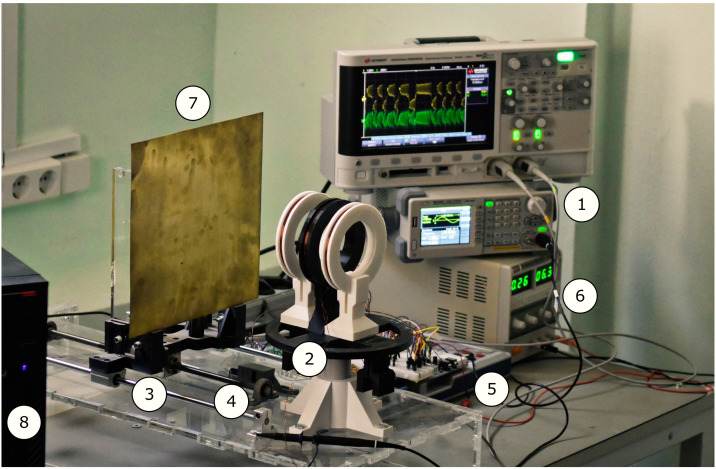
Determination of the largest detection distance by using an automated test bench. Here: (1) sine wave generator and the scope; (2) coils setup; (3) moving cart with the target mounted on it; (4) null position optical sensor; (5) NI ELVIS III device for interfacing and powering the metal detector circuitry and the bench; (6) power supply of the stepper motor of the bench; (7) the targe –square copper plate with 25 cm edge; and (8) personal computer for data acquisition and control.

**Figure 18:**
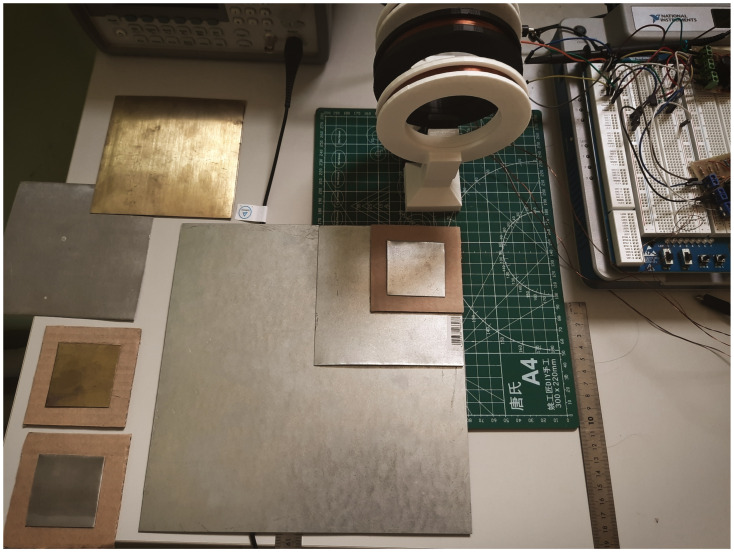
Targets: square metal plates of steel, aluminium and brass of sizes 25 × 25 cm, 12 × 12 cm and 5 × 5 cm.

Figure 17 demonstrates the course of the experiments. Theaccuracy of target positioning at the automated test bench is approximately 50 µm, resulting in a relative error not more than 0.2% in linear displacement measurements [15]. Figure 18 presents the photograph of the used set of targets. We used plates of steel, aluminum and brass of three different sizes.

**Figure 19:**
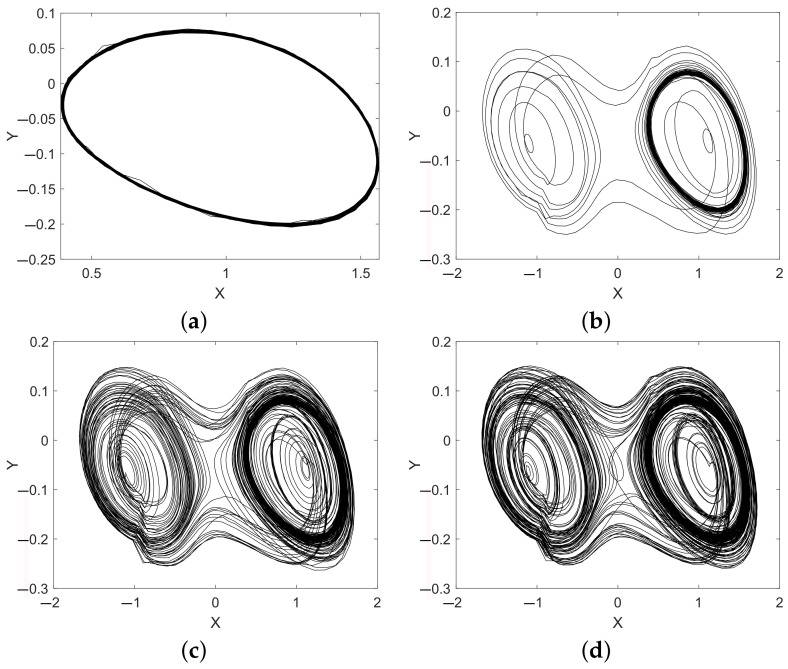
The Duffing oscillator attractors acquired from the sensitive circuit at different distances between the sensing coil and the square 25 × 25 cm brass plate: (**a**) 28 cm; (**b**) 27 cm; (**c**) 24 cm; and (**d**) 20 cm.

A set of chaotic attractors observed as the big (25 × 25 cm) brass plate is getting close to the coil is presented in Figure 19. This example is considered in detail because for this target the detection distance turned out to be the largest.

**Figure 20:**
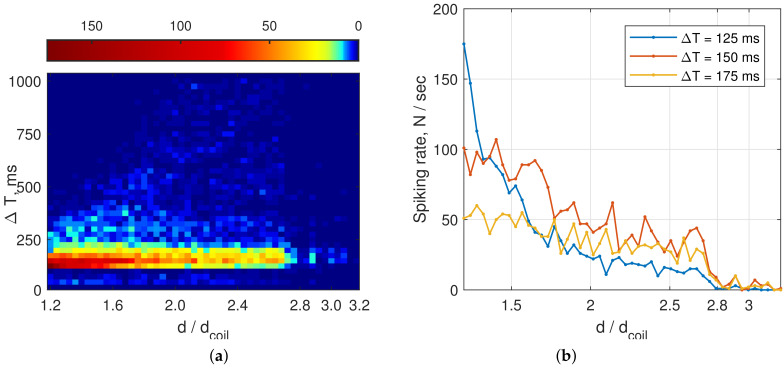
Spiking rate vs. distance between target (square brass plate 25×25 cm) and the closest coil: (**a**) interspike distribution diagram; and (**b**) the most intense spiking intervals.

Intersipke intervals diagrams of the Duffing oscillator are presented in Figure 20. One may see that, though attractors at 20 cm and 24 cm distances look the same (Figure 19c,d), the histogram analysis shows a clear and significant difference between these two distances (corresponding to values 2.0 and 2.4 at *X* axes).

**Figure 21:**
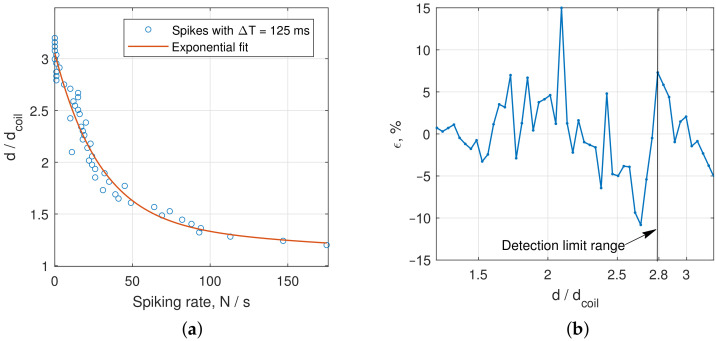
Determination of the distance to the brass plate on the count of interspike intervals with ΔT=125 ms: (**a**) experimental data curve fitting; and (**b**) distance detection error relative to the limit distance d=2.8⌀. Obtained approximation may be used for practical distance measurements.

Figure 21 shows results of the approximation of the distance vs. spikes with ΔT=125 ms count *N* with the following expression:

(10) $$d=1.695e^{-0.03378N}+1.358e^{-6.34\cdot 10^{-4}N}.$$

As one can see in Figure 21b, the range detection error ϵ mostly does not exceed 5% up to distance d=2.5⌀, which is times higher than the accuracy of other inductive sensors at long ranges. At distances d≤1.5⌀, the approximation accuracy does not exceed 2%, which is sufficient for most industrial and aerospace applications. The measurement time was 1 s in each distance position, with the mean frequency of the oscillations in the circuit of about 1700 Hz.

After examining all the targets, we have obtained the following detection distances, see Table 1. Note that all the values are natural numbers for ease of interpretation. As one may see from Figure 20, the strict determination of the sensor limit distance requires an introduction of a certain criterion, as it is a somehow vague value.

For ease of generalization of the results, we converted the target sizes into relative values proportional to the coil diameter—see Table 2. Recall that all the coils in our experiments have a diameter of 10 cm.

### 3.4. Comparison of the Differential Amplifier Signal Analysis by the Duffing Oscillator and Conventional Numerical Analysis

To show that the the Duffing oscillator has an extremely high sensitivity, we analyzed the differential amplifier (DA) output with well-known digital signal analysis methods: spectral analysis, short-time Fourier transform analysis and return map analysis; i.e., we analyzed numerically the same signal which was fed to the analog the Duffing oscillator circuit. It was acquired using the NI ELVIS III station with a sample rate of 68 kHz and resolution of 16 bit between −10 V to 10 V values.

#### 3.4.1. Frequency–Domain Analysis

**Figure 22:**
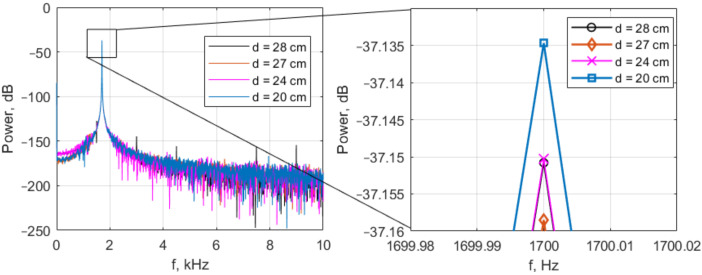
Spectra of differential amplifier outputs at different distances. One may see that only at distance d=20 cm does the signal energy slightly increase, which indicates that the differential amplifier has detected the receiving coils unbalancing.

Figure 22 shows the spectral analysis of the differential amplifier output.

**Figure 23:**
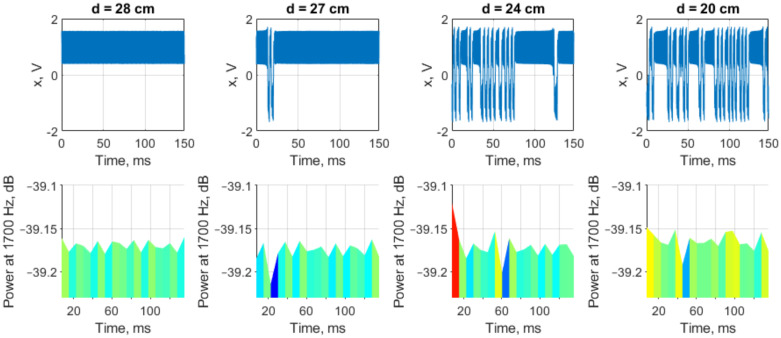
STFT analysis of the differential amplifier output (lower row) and corresponding Duffing oscillator time-domain output (upper row). Compared with the case *d* = 28 cm (target not recognized), the spectrogram at d=20 cm clearly shows an increase in the signal energy at a harmonic oscillator driving frequency of 1700 Hz. However, at longer distances, the signal energy briefly or even imperceptibly exceeds the idle state energy.

The results show that conventional Fourier analysis is not comparable in sensitivity to the Duffing oscillator analysis. There is a way to increase the sensitivity of the Fourier analysis by applying the division of the segments of the signal under study in time—the so-called short-time Fourier transform (STFT). Figure 23 shows outputs of the Duffing oscillator and the corresponding energy of the DA signal at a peak frequency of 1700 Hz (transmitting coil driving frequency), taken for 150 ms. No relationship between fluctuations of DA signal energy at a frequency of 1700 Hz and the attractor of the Duffing oscillator scroll switching was found. However, it is clear that the energy of the DA signal is not a constant value, and STFT analysis is preferable to simple Fourier analysis. We assume that the lack of sensitivity of the STFT analysis is associated with insufficient signal time resolution or insufficient duration.

Assuming that the average signal energy at 1700 Hz changed from −39.18 dB to −39.17 dB as the target approached from 27 to 20 cm (i.e., by 0.12% in a linear scale), the Duffing oscillator has a sensitivity of at least −58 dB. In practice, this number is obviously less, and the estimate of −67 dB found in [7] sounds realistic.

#### 3.4.2. Time-Domain Analysis

As the time-domain method, we applied the return map analysis. It had been developed by Perez and Cerdeira in 1995 [21] for attacking chaotic communication systems. The papers [22,23,24] describe improvements and applications of return map for advanced signal analysis. All the above indicate that return maps can be effectively used to find the difference between the investigated signals in the presence of a small difference between them.

The return map technique assumes a simple transformation of the local minima ($Y_{m}$) and maxima ($X_{m}$) values of the input signal, which can be estimated by the following variables:

(11) $$\begin{array}{c} A_{m}=\frac{X_{m}+Y_{m}}{2};\;\;B_{m}=X_{m}-Y_{m};\;\;C_{m}=\frac{X_{m+1}+Y_{m}}{2};\;\;D_{m}=Y_{m}-X_{m+1}. \end{array}$$

These are the average values of a consecutive maximum–minimum pair and their distance. One may see that they have the exact inversion symmetry so that the *A* vs. *B* section is identical to the −C vs. −D section.

**Figure 24:**
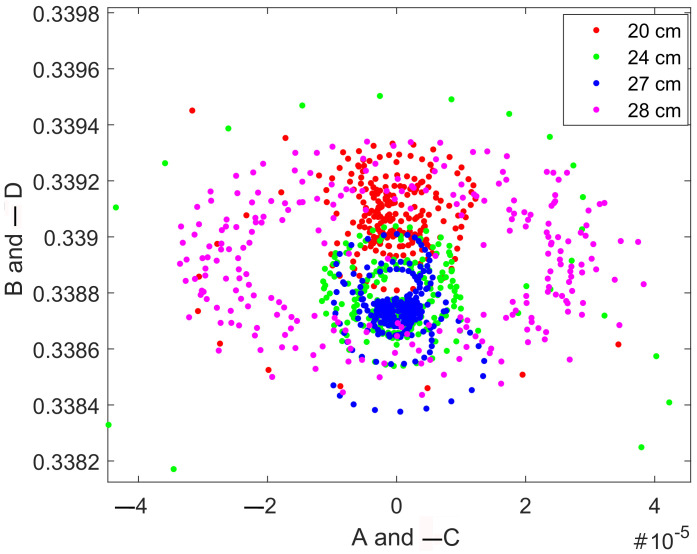
Return map analysis of the differential amplifier output. One may see the certain difference between signals, but still it is hard to quantify.

Figure 24 presents a return map analysis of the differential amplifier signals. The difference between signals may be observed, though it is still very small—note the order of magnitude in the axes is hard to interpret, in contrast to spike analysis of the Duffing oscillator, which offers a simple and accurate way of converting measurements to distances.

## 4. Discussion

### 4.1. Practical Applicability of the Developed Metal Detector

#### 4.1.1. Stability of the Sensor Behavior

In our experiments, we found that when using a stable generator of the harmonic waveform, the analog part of the metal detector also behaves stably, providing, after adjusting the sensitivity, the minimum number of false detections for a long time. At least, the stability of the circuit was found to be much higher than in the study of a metal detector based on the Sprott Case N system [15]. We believe that this is one of the reasons why the sensitivity of the Duffing oscillator-based detector is significantly higher. However, the detector is still difficult to set up to detect targets at large distances. The procedure includes tuning the millivolts of the harmonic wave generator amplitude, and the stability of operation of the detector in these conditions still lasts an impractically short time. An auto-tuning of the differential amplifier gain and numerical implementation of the Duffing oscillator in the digital detector unit may be a proper solution.

#### 4.1.2. Possible Sensitive Coil Setups

**Figure 25:**
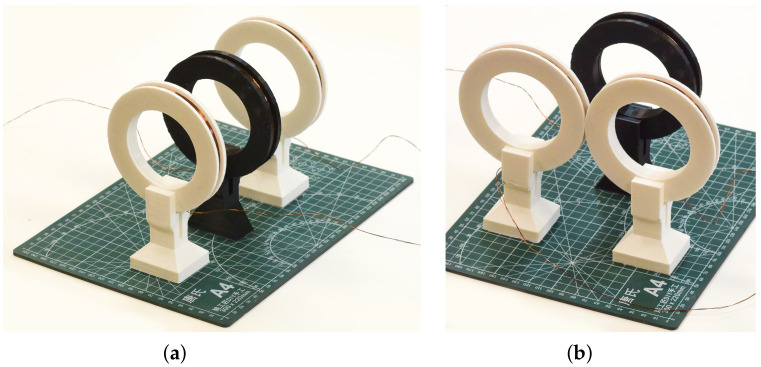
Coils setup: (**a**) in-line, as proposed in [8]; and (**b**) V-shape. The black coil is a transmitting coil, and the white coils are receiving coils.

In most applications, the three-coil design proposed by W. Hu and Z. Liu for the conveyor detector is redundant. We see the following possibilities to apply it to other tasks. We found that the coil setup shown in Figure 25a is bidirectional; when a metal target approaches one of the receiving coils, the differential amplifier signal increases, and when the target approaches the other, it decreases. To make this detector work as proposed, at the edge of periodic and chaotic modes, it is necessary to find a narrow band of periodic oscillations on the bifurcation diagram. A value of A = 0.39 V at 1700 Hz could be a good candidate, as shown in the Figure 13 and Figure 14. Alternative coil setups can also be suggested. For example, the configuration in Figure 25b may be useful for a mine detector or a door frame detector with an extended unidirectional field of action.

### 4.2. Sensitivity Comparison of the Developed Detector with Other Sensor Designs

One of the objectives of the work was to compare the achieved results in the detection range with the performance of metal detectors and inductive proximity sensors described in the scientific literature. In Table 3, we list the largest relative target detection distances of the competing sensors. Though our design detected steel and aluminum targets at closer distances due to the higher conductivity of these metals compared to brass (copper), the largest detection range of our experiments was 2.22 times higher than that of the conventional sensor, and 2 times higher than for the sensor based on chaotic Sprott Case N oscillator utilizing a single coil.

Let us briefly consider designs listed in Table 3. In [25], the authors present an industrial inductive proximity sensor with an analog–digital processing unit suitable for temperature compensation. The use of a digital part allows reducing the influence of analog circuit inaccuracies. In [26], the inductive proximity planar sensor with wireless data readout is described; the sensor works at frequencies up to 30 MHz and distances of 1–15 mm. In [27], a novel multilayer planar coil is connected to the industrial ECS75 eddy current probe conditioner, and the measurement capabilities of this proximity sensor design are investigated. In [29], authors examine low-temperature co-fired ceramics (LTCC) coil equipped with on-the-shelf chip LDC1000 for inductance-to-distance conversion. The last paper in the comparison, ref. [15], is our previous work on the chaos-based metal detector/proximity sensor. The circuit implements a Sprott Case N oscillator and is equipped with a 130 µH single-layer planar coil. A remarkable feature of all of these studies is that sensors there are used for detecting large planar targets. This makes the relative comparison of them with each other and with the sensor developed in the current study possible, as the sensing distance of the inductive sensor in this case is almost completely defined by the coil size (although the geometry and materials of the coil also matter).

**Figure A2:**
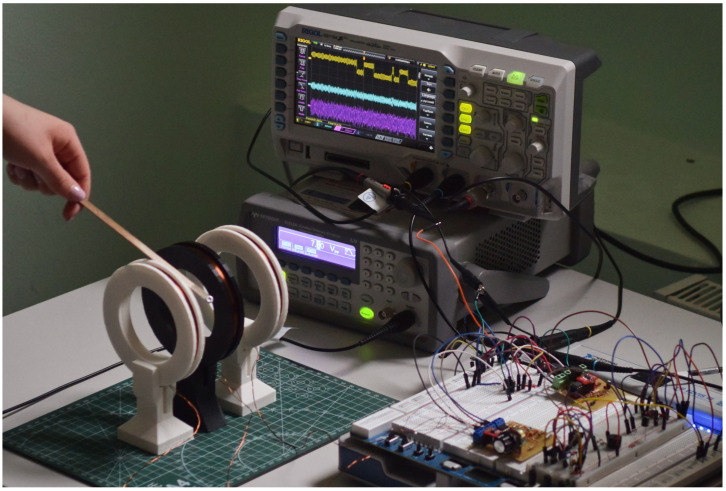
Detection of M2.5 steel washer with 5 mm outer diameter and 0.5 mm thickness. The washer in mounter on a wooden holder, and may be detected if placed in between coils winding; distance between coils is 40 mm.

In the course of the experiments, we examined our sensor’s ability to detect small metal objects, such as an M2.5 washer, see Figure A2. However, it is difficult to find comparative data on the ability of various detectors to sense small objects in the literature, and the comparison as in Table 3 was not performed.

## 5. Conclusions

In the current study, the findings and conclusions are as follows.

1. We proposed a definition and an algorithm for calculating the sensitivity of chaotic oscillators in the context of their application for sensor systems.
2. We have assembled a metal detector consisting of a transmitting coil, two similar receiving coils connected to a differential amplifier, the circuit implementing Duffing oscillator, and a sound indicator. Using the proposed algorithm, we investigated the developed electronic circuit sensitivity and found that its highest value can be achieved at the point of rapid switching from the periodic mode to the chaotic mode at the Duffing oscillator input sine wave amplitude A=169 mV at frequency f=1700 Hz.
3. To numerically estimate the distances between sensitive coils and target from the waveform of the Duffing oscillator, we counted the number *N* of switching between the scrolls of the double-scroll Duffing attractor. We found that at distances to the target from 1.2 to 1.5 of coil diameter, the error of the exponential approximation of *N* did not exceed 2%, which is sufficient for many demanding applications, and at distances up to maximum detection range, it averaged about 5%.
4. We managed to detect metal plates at distances up to 2.8 times coils diameter, depending on the material. We have compared the obtained results with available data on inductive sensors and metal detectors and found that this is the state-of-the-art result.

Moreover, we have recorded and analyzed the signals, normally recognized by the Duffing oscillator in our design, with some digital time- and frequency-domain signal-processing techniques. Though we found a slight difference in the recordings by the digital processing, we concluded that interpretation of these signals provided by the Duffing oscillator is measurable much easier. Thus, the circuit under study may be used for detecting metal objects at long distances, as well as for a variety of other technical applications.

For further research, we are intending to design a metal detector in which not only amplitude but also frequency changes before drive the Duffing oscillator. We believe this would make a metal detector applicable for metal selection. The developed algorithm will be used in the study of other chaotic systems, including oscillators with double-scroll attractors.

## Figures and Tables

**Table 1:** Detection distances for plates of different metals and sizes. All dimensions are in cm.

Plate Size	Steel	Brass	Aluminum
25 × 25	22	28	25
12 × 12	10	15	14
5 × 5	1	7	7

**Table 2:** Detection distances for plates of different metals and sizes in terms of sensitive coils diameter.

Plate Size	Steel	Brass	Aluminum
2.5 × 2.5	2.2	2.8	2.5
1.2 × 1.2	1.0	1.5	1.4
0.5 × 0.5	0.1	0.7	0.7

**Table 3:** Comparing the proximity range of the developed sensor with the sensors in referred works.

Sensor	Max. Range/Coil Width
Cylinder coil and mixed analog-digital unit [25]	0.66
Passive Wireless LC Sensor Based on LTCC [26]	0.75
Multilayer PCB coil and ECS75 conditioner [27]	1
Inductive Proximity Magnetoplated Wire Sensor [28]	1
LTCC planar coil and TI LDC1000 chip [29]	1.26
Single-coil chaotic metal detector [15]	1.4
Current study	2.8

## Data Availability

Not applicable.

## Abbreviations

The following abbreviations are used in this manuscript:

BD	Bifurcation diagram
DA	Differential amplifier
DC	Direct current
LLE	Largest Lyapunov exponent
LTCC	Low temperature co-fired ceramics
LUT	Look-up table
NI	National Instruments (Austin, TX, US)
PCB	Printed circuit board
RMS	Root-mean-square
RQA	Recurrence quantification analysis
STFT	Short-time Fourier transform
TI	Texas Instruments (Austin, TX, US)

## Appendix A

The figures below explain the operation of a differential amplifier in a metal detector based on a Duffing oscillator.
